# Automated Bot Detection Using Bayesian Latent Class Models in Online Surveys

**DOI:** 10.3389/fpsyg.2022.789223

**Published:** 2022-04-27

**Authors:** Zachary Joseph Roman, Holger Brandt, Jason Michael Miller

**Affiliations:** ^1^Department of Psychology, University of Zurich, Zürich, Switzerland; ^2^Department of Psychology, Faculty of Mathematics and Natural Sciences, University of Tübingen, Tübingen, Germany; ^3^Department of Psychology, University of Kansas, Lawrence, KS, United States

**Keywords:** latent class analysis, mixture models, structural equation models, MTurk, bots

## Abstract

Behavioral scientists have become increasingly reliant on online survey platforms such as Amazon's Mechanical Turk (Mturk). These platforms have many advantages, for example it provides ease of access to difficult to sample populations, a large pool of participants, and an easy to use implementation. A major drawback is the existence of bots that are used to complete online surveys for financial gain. These bots contaminate data and need to be identified in order to draw valid conclusions from data obtained with these platforms. In this article, we will provide a Bayesian latent class joint modeling approach that can be routinely applied to identify bots and simultaneously estimate a model of interest. This method can be used to separate the bots' response patterns from real human responses that were provided in line with the item content. The model has the advantage that it is very flexible and is based on plausible assumptions that are met in most empirical settings. We will provide a simulation study that investigates the performance of the model under several relevant scenarios including sample size, proportion of bots, and model complexity. We will show that ignoring bots will lead to severe parameter bias whereas the Bayesian latent class model results in unbiased estimates and thus controls this source of bias. We will illustrate the model and its capabilities with data from an empirical political ideation survey with known bots. We will discuss the implications of the findings with regard to future data collection *via* online platforms.

## 1. Introduction

In the behavioral sciences, online survey platforms (e.g., Amazon's Mturk) are a common method of data collection. They provide an affordable means of collecting large amounts of data from potentially difficult to obtain populations in a short period of time. Recently, a major drawback of the approach has come to light, exploitation of the framework for monetary gain. This is achieved in part by “click farms” where people indiscriminately complete surveys, or by malicious software which does the same. We generally refer to both of these forms of data contamination as “bots.” Chmielewski and Kucker (2020) conducted a longitudinal survey study (four waves of data from 2015 to 2019) using Mturk which established that a substantial decrease in data quality occurred over the duration of the study. This evidence suggests that around this time bot frequency was on the rise. In response, online survey platforms have implemented more stringent screening criteria. However, incentives produce innovations. As platforms and researchers introduced approaches to screen for bots, those who aim to profit continue to adapt. For example, Sharpe Wessling et al. (2017) points out that forums exist in which people share approaches and software to bypass screening criteria.

### 1.1. Identifying Inattentive Behavior in the Literature

The bot problem is relatively new, however literature on inattentive human responders is well-established (e.g., Wise and DeMars, 2006; Huang et al., 2012, 2015; Huang and Liu, 2014; DeSimone et al., 2015; Dupuis et al., 2019). In both scenarios, researchers aim to identify response sets which are not representative of deliberate responses. While these situations are different, we find it useful to borrow insight from inattention literature to inform approaches on identification of bots (Meade and Craig, 2012).

Bots reduce the quality of data collection. Bots do not respond to surveys in line with instructions. Therefore, we can think of their contribution as random noise (Meade and Craig, 2012; Buchanan and Scofield, 2018). The presence of random noise increases error variance and pulls item correlations toward zero. Consequently, type II error rates are inflated and scale creation and validation is detrimentally affected (Marjanovic et al., 2014).

Methods developed to identify inattentive response behavior can be classified into three categories. Methods from the first category utilizes external details like bogus items, validity scales, or response times (e.g., Wise and DeMars, 2006; Huang et al., 2012, 2015; Huang and Liu, 2014; DeSimone et al., 2015). Second, a set of approaches is used to calculate indices based on the response pattern. For example, Greene (1978) identified mismatched responses for positively and negatively worded items, Baumgartner and Steenkamp (2001) and Baumgartner and Steenkamp (2006) calculated frequencies of participant responses of the same category (e.g., frequency of endorsing “strongly agree”). Furthermore, person-fit-indices (Drasgow et al., 1985; Karabatsos, 2003), and outlier-based approaches (Curran, 2015; DeSimone et al., 2015) exist. These approaches all share a common procedure: First an index is calculated and the inattentive responders are identified for falling above or below a predetermined threshold; the identified responders are removed from the sample; statistical analysis are then conducted on the remaining data.

The third category includes statistical methods that incorporate the detection of inattentive persons in the actual analysis (e.g., a confirmatory factor analysis; CFA). The majority of these approaches utilize the latent class framework (i.e., mixture modeling) and directly model inattentive patterns (Meade and Craig, 2012; Terzi, 2017; Jin et al., 2018). In these analysis, persons are grouped into two or more classes, where one class includes respondents who answer according to the instructions, and the remaining classes model alternative response patterns that are independent of the item content such as responding randomly. Identification of the latent classes is based on a specific model that reflects these expected patterns such as uniform probabilities for all answers (Jin et al., 2018). Alternatively, they use external information, for example, fit indices calculated a priori (e.g., outlier measures; Terzi, 2017).

### 1.2. Bayesian Latent Class Models for Identification of Inattentive Behavior

Bayesian Markov Chain Monte-Carlo (MCMC) estimation has many benefits over frequentist approaches, which in conjunction with technological advances (e.g., increased computer memory, processing, and ease of parallel processing) have lead to an increase in their popularity among behavioral sciences (Van de Schoot et al., 2017). Bayesian MCMC estimation allows for flexible and complex specifications (e.g., Lee et al., 2007; Muthén and Asparouhov, 2012; Roman and Brandt, 2021), in addition to fewer estimation issues (e.g., Depaoli and Clifton, 2015) and lower sample size requirements (e.g., Hox et al., 2012). Further, joint modeling approaches allow Bayesian models to simultaneously sample missing values or latent scores while estimating parameters of a model of interest (Dunson et al., 2003). This makes Bayesian estimation an ideal choice for estimating latent class models in a joint approach with a model of interest.

Jin et al. (2018) conducted a series of Monte-Carlo studies which explored the performance of Bayesian latent class models to identify inattentive respondents in an item response setting. The authors varied the percent of inattentive responders (0, 10, 20, and 30%), test length (10 and 20 items), and inattentive response pattern (only middle category and random), among other conditions. The latent class model performed well in the high inattention condition of 30% random responses, and importantly, in the 0% random response condition. Further, an intuitive yet important finding is the impact of test length on identification. When test length was 10 items correct classification was 83.47%, at 20 items this improved to 95.38%, this suggests there is a minimum number of items necessary to identify a random response pattern accurately. Jin et al. (2018) provides a successful example of the ability for latent class models to identify aberrant response patterns related to inattention. For latent variable models using the CFA framework, such an approach has not yet been tested. It is also unknown so far how well such procedures work for the identification of bots. Here, we will use a LC-CFA to identify such bots.

### 1.3. Scope and Outline

The remainder of the manuscript focuses on the automated detection of bots using latent class confirmatory factor analysis (LC-CFA). We will introduce a generalized approach for factor analytic methods that cover continuous, binary, or count data. We will provide information on how to identify a latent class consisting of bots. We will illustrate in a simulation study that the model has optimal performance under a variety of different empirically relevant scenarios. We will also show how detrimental bots are for the performance of standard CFA methods. Using empirical data with known bots, we will evaluate model performance in the context of an Amazon Mturk study which collected political survey data.

The next section includes the model formulation and its Bayesian implementation. Then we provide the simulation study design and results. Followed by the empirical example, before we discuss the feasibility of the approach, its limitations, and future directions.

## 2. Bayesian Latent Class Model

In this section, we provide a general model formulation for the LC-CFA that can be used to detect bots in online questionnaires. The model is comprised of two latent classes: The first class *C* = 1 includes persons who provide responses according to a factor model assumed to underlie the items. The second class includes bots who do not provide information but instead choose random responses. Class membership is modeled using a confirmatory method based on both a specific model for random responses, and logistic model that uses indices based on person-fit measures to detect non-respondence.

For each item *Y*_1_, …, *Y*_*j*_, a general measurement model is formulated for *i* = 1…*N* in class *C* = 1 (valid responses) and *C* = 2 (bots) by:


(1) $$\begin{array}{l} g\left(\mu_{y,ij}{|}_{C_{i}=1}\right)=\tau_{j1}+\lambda_{j}\eta_{i} \end{array}$$

(2) $$\begin{array}{l} g\left(\mu_{y,ij}{|}_{C_{i}=2}\right)=\tau_{j2} \end{array}$$

(3) $$\begin{array}{l} Y_{ij}{|}_{C_{i}=c}\sim F\left(\mu_{y,ij}{|}_{C_{i}=c},\left(\sigma_{y,jc}^{2}\right)\right) \end{array}$$

where *g* is a link function and *F*[μ, (σ^2^)] is a distribution function with mean μ and dispersion related parameter σ^2^, which is necessary for some distributions. For example, for binary items, the link function is a logit function and the distribution function is the Bernoulli distribution. For continuous items, an identity function is used as link and a normal distribution function is used. And for count items, a log link function can be used with a Poisson distribution function (details on generalized models can be found in, e.g., Song et al., 2013; Wood, 2017).

*C*_*i*_ is a latent categorical variable indicating if a participant is flagged as a bot (*C* = 2) or a person providing meaningful information (*C* = 1), i.e., responses in line with the factor model. τ_*jc*_ is a class-specific intercept for item *j*, ***λ***_*j*_ is an *m* dimensional vector of factor loadings on the *m* factors $\eta ={\left(\eta_{1},\ldots ,\eta_{m}\right)}^{\prime }$ in class *C* = 1. For normal distributions, error variances are assumed to be state-specific (i.e., $\sigma_{y,jc}^{2}$).

For the latent factors in class *C* = 1, we assume


(4) $$\begin{array}{l} \eta_{i}{|}_{C_{i}=1}\sim MVN\left(\kappa ,\Phi \right) \end{array}$$

where *MVN* is a multivariate normal distribution with *m* dimensional mean vector ***κ*** and *m* × *m* covariance matrix **Φ**. Other distributions such as the *T*-distribution can be used instead if the construct under investigation is assumed to be non-normal (e.g., Muthén and Asparouhov, 2014). We assume that standard identification constraints for SEM hold with regard to the scaling of the latent factors (e.g., by using a scaling indicator).

### 2.1. Interpretation of Classes

In order to identify the model and ensure that the classes refer to persons vs. bots, specific model restrictions are imposed on the class-specific parameters and a prediction model for the class membership should be used. This idea is in line with recent suggestions about confirmatory uses of latent class models (Jeon, 2019). If these restrictions are not imposed, classes may relate to any kind of differences with regard to distribution or relationships between variables (for similar problems in latent class modeling, see Hipp and Bauer, 2006).

The model formulation for the bots in class *C* = 2 above results in a statistical model that is in line with a random response provided by the bots. For example, for continuous items, an item mean and a variance is used [i.e., $\left(Y_{ij}|C_{i}=2\right)\sim N\left(\tau_{j2},\sigma_{y,j2}^{2}\right)$] to model random responses. For binary and ordinal items, the model formulation results in a logistic or ordinal model with equal probabilities to select either of the categories (for a similar approach for inattentive responses, see Jin et al., 2018).

The prediction of the latent class membership *C*_*i*_ = *c* is specified using a multinomial logistic model based on two indices that can capture the randomness of the responses (e.g., for similar models to predict latent class membership, see Muthén and Asparouhov, 2009; Kelava et al., 2014; Asparouhov et al., 2017):


(5) $$\begin{array}{l} P\left(C_{i}=1|{ϒ}_{1i},{ϒ}_{2i}\right)=expit\left(\beta_{0}+\beta_{1}{ϒ}_{1i}+\beta_{2}{ϒ}_{2i}\right) \end{array}$$

with *expit*(*x*): = 1/(1 + *exp*(−*x*)).

This additional model is used to improve identification of bots *via* an explicit evaluation of the overall response pattern. In order to achieve this, we use person-fit indices that can be calculated based on the response pattern. In contrast to previous uses of person-fit indices, we do not use cut-offs or delete persons by hand. Instead, a model based approach is used here that provides a probability statement for each person to be a bot or not.

Several previous authors used similar latent class models in a Bayesian setting without a direct model for the probability π = [*P*(*C*_*i*_ = 1), …, *P*(*C*_*i*_ = *C*_*max*_)]. That implied that all persons have the same probability to be in classes *C* = *c* because a single unconditional distribution is used. For a Bayesian implementation, this is done *via* the Dirichlet prior, that is π ~ *Dir*(*a*) (e.g., Depaoli, 2013, 2014), where π and *a* are vectors with as many entries as classes (*C*_*max*_) are modeled. In comparison to our model, this approach would be very similar to removing all predictors from the model in Equation (5) and using only β_0_ in the multinomial model (for similar implementations see, e.g., Asparouhov and Muthén, 2010; Asparouhov and Muthén, 2016; Kelava and Brandt, 2019).

### 2.2. Person-Fit Index

ϒ_1_ is a likelihood based person-fit index that has been shown to provide information to detect inattentive persons. This person-fit index can be extracted from the following procedure (Lange et al., 1976; Reise and Widaman, 1999; Terzi, 2017): First conduct a CFA for all persons and extract the model-implied mean vector and covariance matrix (***μ***, **Σ**) to calculate the individual likelihood contributions under the assumption of multivariate normality


(6) $$\begin{array}{l} ll_{i}\left(\mu ,\Sigma \right)=-\frac{1}{2}\left(p\cdot \ln\left(2\pi \right)+\ln|\Sigma |+D_{i}^{2}\left(\mu ,\Sigma \right)\right) \end{array}$$

with a Mahalanobis distances $D_{i}^{2}$ based on these model-implied mean vector and covariance matrix


(7) $$\begin{array}{l} D_{i}^{2}\left(\mu ,\Sigma \right)={\left({\text{y}}_{i}-\mu \right)}^{\prime }\Sigma^{-1}\left({\text{y}}_{i}-\mu \right) \end{array}$$

Calculate


(8) $$\begin{array}{l} {ϒ}_{1i}=-2\cdot \left(ll_{i}\left(\mu ,\Sigma \right)-ll_{i}\left(\overset{-}{\text{y}},\text{S}\right)\right) \end{array}$$

where $ll_{i}\left(\overset{-}{\text{y}},\text{S}\right)$ is the corresponding statistic based on the empirical mean vector and covariance matrix $\overset{-}{\text{y}},\text{S}$. As can be seen from this definition, the person-fit index will provide information about each person's likelihood contribution. More details about its properties can be found in Reise and Widaman (1999)^1^.

While this approach can be used for any type of data with the respective link and distribution function, it has mainly been used for continuous items. Similar and specialized model-based fit indices were independently defined for binary and ordinal data (e.g., Levine and Rubin, 1979; Drasgow et al., 1985; Meijer and Sijtsma, 2001; Snijders, 2001; Terzi, 2017).

### 2.3. Variability Index

In addition, we propose an alternative approach with fewer assumptions to use as ϒ_2_. We do not make any distributional assumptions as they are necessary for the individual likelihood contribution above.

The variability index ϒ_2_ is defined as the averaged factor-specific item variance:


(9) $$\begin{array}{l} {ϒ}_{2i}=\frac{1}{m}\overset{m}{\underset{k=1}{\sum }}Var\left({\text{y}}_{ik}\right) \end{array}$$

where **y**_*ik*_ includes all scores for person *i* of the items that are loading on the *k*-th factor. The logic of this index is as follows: Assuming that the configuration of the factor model holds, responses to items that belong to the same factor should have a rather small variability because persons are more likely to respond in a similar fashion depending on their expression of the construct (e.g., low or high)^2^. Bots with a random response modus will provide a larger variability in comparison.

**Figure 1:**
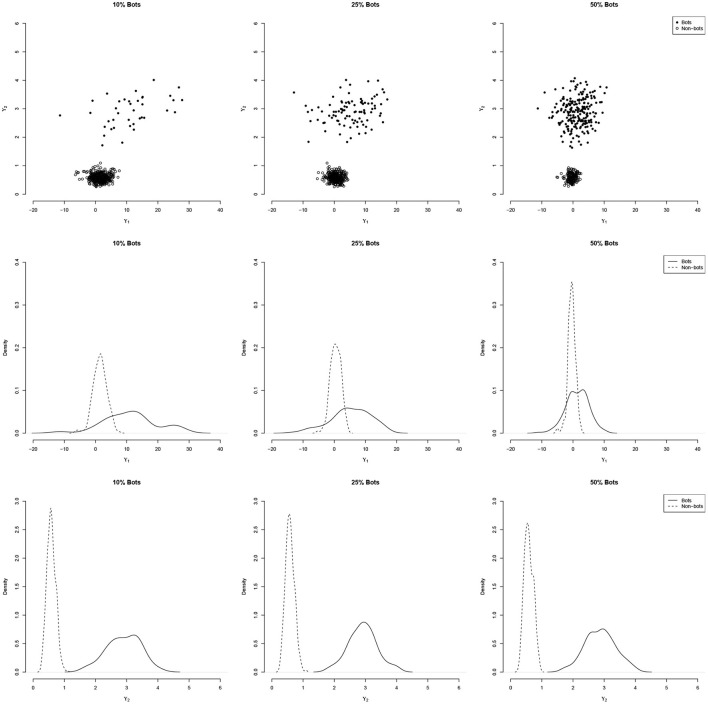
Illustration of the performance of the person-fit index ϒ_1_ and the variability coefficient ϒ_2_ to distinguish bots from Non-bots under different levels of contamination. The **(top)** panels show the bivariate distribution, the **(middle and bottom)** panels show the densities for person-fit index ϒ_1_ and variability coefficient ϒ_2_, respectively. While the distinction between bots and non-bots becomes more difficult with the person-fit index with increasing contamination, the variability coefficient remains unaffected.

Figure 1 illustrates the distribution of two indices ϒ_1_ and ϒ_2_ for a simulated data set with *N* = 400 persons and a six-factor model (see details in the simulation section) with increasing amounts of bot contamination (10, 25, and 50%). As the figure show, the variability coefficient can clearly distinguish the two subgroups.

In comparison to the approach by Jin et al. (2018) who developed a similar model for inattentive behavior, we would like to highlight the following aspects. Jin et al.'s (2018) approach focused on IRT models only (including the Rasch model and generalized partial credit model for ordinal scaled data. Here, we provided a more general approach based on generalized models that include this approach as a special case but also covers continuous and count data. This results in a higher flexibility particularly if models are used for questionnaire data that have sufficient response categories to assume continuous data (Rhemtulla et al., 2012).

Second, the approach by Jin et al. (2018) defines its non-responsive (inattentive) classes *via* a probability pattern of the items (with a categorical distribution). For example, for ordinal data, they suggest equal probabilities for each response category to model random behavior. This makes it necessary to define different non-responsive patterns in separate latent classes (e.g., extreme responses vs. random responses). However, this strategy comes with two disadvantages: First, if many classes are necessary to capture non-responsiveness classes will become small (e.g., with 10 bots/persons) which will result in numerical instabilities. Second, un-modeled non-responsiveness will inflict bias in the responsive (attentive) group as Jin et al. (2018) show in their simulation study.

In contrast, we only model a single class that captures all non-responsive patterns that could be expected of bots. The definition of the second class is conducted using the multinomial logistic model that extracts the specific deviations from responsive behavior *via* pattern-related predictors as shown in Figure 1.

In comparison to other models that focus on cognitive skill tests (e.g., with regard to non-responses, Pohl et al., 2019; Ulitzsch et al., 2020), we do not need additional information such as reaction times to predict the behavior. This is an advantage in many settings where retrieving such information is impossible or at least cumbersome (also on Amazon's Mturk).

### 2.4. Bayesian Model Estimation

In this subsection, we provide details about model estimation using a Bayesian implementation. Bayesian estimation provides a flexible framework that allows to extend the basic model to any kind of more complex structure (Song et al., 2013; Kelava and Brandt, 2014). Here, we specify the LC-CFA with a straightforward implementation based on priors.

The observed variables' distributions can be specified as for continuous data as


(10) $$\begin{array}{l} \left(y_{ij}|C_{i}=c\right)\sim N\left(\mu_{ijc},\sigma_{ijc}^{2}\right),\;i=1\ldots N,k=1\ldots j, \end{array}$$

where *N*(μ, σ^2^) is the normal distribution with mean μ and variance σ^2^. For binary data, the distribution is specified as


(11) $$\begin{array}{l} \left(y_{ij}|C_{i}=c\right)\sim Bern\left(expit\left(\mu_{ijc}\right)\right),\;i=1\ldots N,j=1\ldots p, \end{array}$$

with a Bernoulli distribution *Bern*(π) and probability for *y* = 1 of π. Finally, for count data a model is specified *via*


(12) $$\begin{array}{l} \left(y_{ij}|C_{i}=c\right)\sim Poisson\left(exp\left(\mu_{ijc}\right)\right),\;i=1\ldots N,j=1\ldots p. \end{array}$$

with a Poisson distribution *Poisson*(λ) with event rate λ.

For a CFA model, the multivariate distribution of the latent factors is given by


(13) $$\begin{array}{l} \eta_{i}\sim MVN\left(\kappa ,\Phi \right),\;i=1\ldots N \end{array}$$

The latent class variable follows a Bernoulli distribution


(14) $$\begin{array}{l} C_{i}\sim Bern\left(\pi_{i}\right),\;i=1\ldots N \end{array}$$

with π_*i*_ = *expit*(β_0_ + β_1_ϒ_1*i*_ + β_2_ϒ_2*i*_). Note that in this model differs from latent class models without predictors that use conjugate priors for the probability π_*i*_ = π∀*i*, that is a Dirichlet prior of the form π ~ *Dirich*(**a**) (e.g., Depaoli, 2013, 2014), where **a** is a hyperprior. In contrast, we use priors for the regression coefficients β_*r*_, *r* = 0…2, in the multinomial logistic model defined in Equation (5).

The priors for the model parameters are given by


(15) $$\begin{array}{l} \tau_{jc}\sim N\left(\mu_{\tau 0c},\sigma_{\tau 0c}^{2}\right),\;j=1\ldots p,c=1,2 \end{array}$$

(16) $$\begin{array}{l} \lambda_{jk}\sim N\left(\mu_{\lambda 0j},\sigma_{\lambda 0j}^{2}\right),\;j=1\ldots p,k=1\ldots m \end{array}$$

(17) $$\begin{array}{l} \kappa_{k}\sim N\left(\mu_{\kappa 0k},\sigma_{\kappa 0k}^{2}\right),\;k=1\ldots m \end{array}$$

(18) $$\begin{array}{l} {\text{Ψ}}^{-1}\sim Wish\left({\text{Ψ}}_{0},df_{\text{Ψ}}\right) \end{array}$$

(19) $$\begin{array}{l} \beta_{r}\sim N\left(\mu_{\beta 0r},\sigma_{\beta 0r}^{2}\right),\;r=0\ldots 2 \end{array}$$

where *Wish*() is the Wishart distribution. In the case of continuous items, the prior for the residual variance is given by


(20) $$\begin{array}{l} \sigma_{jc}^{-2}\sim Ga\left(a_{\sigma jc},b_{\sigma jc}\right),\;j=1\ldots p,c=1,2. \end{array}$$

where *Ga*() is the Gamma distribution.

Here, $\mu_{\tau 0c},\sigma_{\tau 0c}^{2}$ etc. are hyperparameters that need to be chosen.

## 3. Simulation Study

In this section, we will present a simulation study that investigates the performance of the LC-CFA to identify bots and to improve estimation of the relevant parameters of the factor model. In addition, we will compare this performance to a standard CFA.

### 3.1. Data Generation

Data were generated for a three- or six-factor model^3^ (fixed design factor A). Each factor was operationalized with *p* = 6 items **Y**, which is a typical length for a scale in psychological research. In the first step, data were generated using the following measurement model for non-bots:


(21) $$\begin{array}{l} {\text{Y}}_{i}^{*}=\tau_{1}+{\Lambda \eta }_{i}+\epsilon_{i} \end{array}$$

where ***τ***_1_ was an intercept vector, **Λ** a factor loading matrix, a multivariate normally distributed latent factor score matrix ***η***_*i*_[η ~ *MVN*(**0**, **Φ**)] and ***ϵ***_*i*_ were the residuals [ϵ ~ *MVN*(0, ***σ***^2^**I**)]. For data generation, intercepts were set to zero. Factor loadings followed a simple structure pattern (i.e., each set of six items only loaded on a single factor). The variances of the latent factors were set to one and the correlations to ρ, which was a design factor (see below). The residual variances in the vector ***σ*****^2^** was chosen such that the resulting variance of ${\text{Y}}_{i}^{*}$ was one; the actual values depended on the chosen communalities (design factor D).

(Non-zero) standardized factor loadings were randomly chosen from a uniform distribution around an average item communality for each replication in a range of $\sqrt{Communality\pm 0.15}$. Average communalities were included as a random design factor D that was sampled for each replication from a uniform distribution ranging from 0.25 to 0.64. These values covered typical item communalities encountered in psychological research (Chaplin, 1991; Kelava and Nagengast, 2012).

The latent factors were normally distributed with an intercorrelations randomly sampled from a uniform distribution lying between 0.0 and 0.7 (random design factor E). This again covered a typical range of multicollinearity from completely uncorrelated to highly correlated factors. All latent item scores $Y_{i}^{*}$ were standard normally distributed (i.e., zero mean and variance one).

In a second step, item scores for the non-bots (*C*_*i*_ = 1) were generated on a six-point Likert-style scale ranging from 1 through 6 using a standard threshold function with equidistant steps:


(22) $$\begin{array}{l} \left\{Y_{i}=k|C_{i}=1\right\}=\delta_{k-1}\leq \left\{Y_{i}^{*}|S_{i}=1\right\}<\delta_{k} \end{array}$$

with δ = (−∞, −2, −1, …, 2, ∞). For the bots (*S*_*i*_ = 2), a completely random pattern was assumed


(23) $$\begin{array}{l} Y_{i}{|}_{C_{i}=2}\sim Cat\left(\pi \right) \end{array}$$

with a categorical distribution *Cat*() and ***π*** = (1/6, …1/6).

Data were generated for sample sizes of *N* = 200, 400, and 800 (fixed design factor B). The percentage of bots were set to 10, 25, and 50% (fixed design factor C). This resulted in 2 × 3 × 3 = 18 fixed design conditions. *R* = 500 replications were generated under each condition of the fixed design factors. Table 1 summarizes the simulation conditions.

**Table 1:** Simulation design with three fixed (A, B, C) and two random (D, E) factors.

**Factor**	**Label**	**Levels**		
A	Model complexity	3 factors	6 factors	
B	Sample size	200	400	800
C	Percentage bots	10%	25%	50%
D	Communality λ^2^	0.25	to	0.64
E	Factor correlations ρ	0.00	to	0.70

### 3.2. Data Analysis

For the analysis, two models were specified: A standard Bayesian CFA model and a LC-CFA model. Details on the LC-CFA and its implementation can be found in the methods section. The CFA model was identical to the model specified for *C* = 1 of the LC-CFA (and did not include mixtures) including the priors.

Priors for parameters were chosen as weakly informative priors using the following hyperparameters:


(24) $$\begin{array}{l} \tau_{jc}\sim N\left(0,1\right),\;j=1\ldots p,c=1,2 \end{array}$$

(25) $$\begin{array}{l} \lambda_{jk}\sim N{\left(0,1\right)}^{+},\;j=1\ldots p,k=1\ldots m \end{array}$$

(26) $$\begin{array}{l} {\text{Ψ}}^{-1}\sim Wish\left({\text{I}}_{m},m\right) \end{array}$$

(27) $$\begin{array}{l} \beta_{r}\sim N\left(0,10\right),\;r=0\ldots 2 \end{array}$$

(28) $$\begin{array}{l} \sigma_{jc}^{-2}\sim Ga\left(9,4\right),\;j=1\ldots p,c=1,2. \end{array}$$

The latent factor means κ_*k*_ were set to zero for identification, in addition the first factor loadings for each factor was constrained to one. **I**_*m*_ was an *m* × *m* identity matrix. We follow the advice of Song et al. (2013) in our choice of hyperparameters Ga(9,4) for the variances $\sigma_{jc}^{-2}$^4^.

We utilized truncated normal priors for the factor loadings λ_*jk*_ for convenience in computational time for the simulation study^5^.

Performance of the LC-CFA was assessed with convergence, bias, and accuracy statistics sensitivity and specificity. First, convergence was assessed with $\hat{R}$ as computed by Gelman et al. (2013). The following indices were based only on estimates from models which reached acceptable convergence criteria. Percent bias is computed as the estimates percent deviation from the population value. Sensitivity is conceptualized as the true positive rate (bot detection rate), and specificity as the true negative rate (non-bot detection rate). Sensitivity is computed as $\frac{TP}{TP+FN}$ and specificity as $\frac{TN}{FP+TN}$ where *TP* is the number of true positive identifications, *TN* is the number of true negative identifications, *FN* is the number of false negative identifications, and *FP* is the number of false positive identifications.

All models were implemented in Jags 4.2 (Plummer, 2003) with three chains and 12,000 iterations each. The first 6,000 iterations were discarded as burn-in. Convergence was monitored for all parameters using the $\hat{R}$ statistic and a cut-off value of $\hat{R}$ <1.01 in line with the advice of Vehtari et al. (2021).

## 4. Results

### 4.1. Convergence

Convergence rates for the CFA were above 99.8% across all conditions (which was expected due to the simplicity of the model). For the LC-CFA, convergence rates depended on sample and model size as well as the proportion of bots as depicted in Table 2. For small sample sizes (*N* = 200) and small model size (*q* = 3 factors), convergence rates were above 88.0%. For small sample sizes (*N* = 200) and large model size (*q* = 3 factors), convergence rates were lower and lay between 30.3 and 61.8%. For larger sample sizes, convergence rates were above 85.0% (*N* = 400) and above 98.4% (*N* = 800). This indicates that the more complex model with six factors needed at least a sample size of *N* = 400 to perform reliably (i.e., converge) under the conditions (e.g., chain length) in this simulation study.

**Table 2:** Convergence rates as well as sensitivity and specificity for the recovery of class memberships for the LC-CFA across conditions of sample size (*N*), number of factors (*q*), and proportion of bots.

***q***	**Proportion bots**	**Convergence**	**Sensitivity**	**Specificity**
***N* = 200**				
3	0.10	88.0	0.96	0.98
3	0.25	94.6	0.99	0.98
3	0.50	97.6	0.99	0.97
6	0.10	61.8	0.99	1.00
6	0.25	54.7	1.00	1.00
6	0.50	30.3	1.00	1.00
***N* = 400**				
3	0.10	91.4	0.99	0.98
3	0.25	98.8	0.99	0.98
3	0.50	99.8	0.99	0.98
6	0.10	85.0	1.00	1.00
6	0.25	96.4	1.00	1.00
6	0.50	99.8	1.00	1.00
***N* = 800**				
3	0.10	99.6	0.99	0.99
3	0.25	100.0	0.99	0.98
3	0.50	100.0	0.99	0.98
6	0.10	98.4	1.00	1.00
6	0.25	100.0	1.00	1.00
6	0.50	100.0	1.00	1.00

### 4.2. Class Recovery

Table 2 also includes the average sensitivity and specificity to identify the bots. Both indices were close to one across all conditions with a minimum average sensitivity of 0.96 (*N* = 200, *q* = 3, 10% bots) and a minimum average specificity of 0.97 (*N* = 200, *q* = 3, 50% bots). These values were independent of the fixed effect design factors. This indicated a very reliable identification of the bots.

### 4.3. Parameter Bias

Table 3 shows the average parameter bias both for the LC-CFA and the CFA. In the table, we present results for the factor variances (ϕ_*jj*_) averaged across factors, the factor correlations (ϕ_*kj*_) averaged across all mutual correlations, and the standardized factor loadings (λ) averaged across all factor loadings.

**Table 3:** Average parameter bias for the LC-CFA and the CFA across conditions of sample size (*N*), number of factors (*q*), and proportion of bots.

**q**	***N***	**LC-CFA**			**CFA**		
		**ϕ_*jj*_**	**ϕ_*kj*_**	***λ***	**ϕ_*jj*_**	**ϕ_*kj*_**	***λ***
**10% Bots**							
3	200	−0.2	7.0	0.8	−12.3	−4.6	−11.5
3	400	−1.6	0.9	−1.1	−12.7	−3.5	−13.5
3	800	−1.5	−0.5	−2.1	−12.8	−3.4	−14.5
6	200	−2.1	9.0	0.9	−14.1	1.0	−10.6
6	400	−4.8	2.1	−1.8	−15.6	−0.8	−13.3
6	800	−3.5	−0.8	−2.8	−13.7	−1.9	−14.6
**25% Bots**							
3	200	0.4	11.7	1.9	−24.6	−13.6	−24.7
3	400	−1.7	1.0	−0.8	−25.9	−7.2	−27.4
3	800	−1.5	−0.5	−1.9	−27.2	−11.1	−28.8
6	200	2.2	21.3	2.8	−27.9	−7.6	−24.3
6	400	−4.6	2.1	−1.2	−30.4	−5.3	−27.5
6	800	−3.7	−0.2	−2.6	−28.8	−4.5	−28.9
**50% Bots**							
3	200	7.0	38.8	5.2	−39.1	−30.8	−42.5
3	400	−0.4	11.1	0.6	−44.0	−31.3	−46.2
3	800	−1.8	5.1	−1.2	−47.7	−18.8	−47.9
6	200	438.1	133.2	21.7	−44.2	−32.3	−42.8
6	400	−2.8	9.0	0.5	−48.4	−17.7	−46.4
6	800	−4.2	−2.8	−1.9	−49.9	−13.7	−48.2

For the LC-CFA, estimates were fairly unbiased for sample sizes above 200 with values ranging between −4.2 and −0.4% for the variances, −2.8 and 11.1% for the correlations, and −2.8 and 0.6% for the factor loadings. Slightly higher values for the correlations were observed under the condition of 50% bots and sample size of *N* = 400, that is, when the number of valid persons providing information for the parameters was only 200 (under *S* = 1).

The performance under the small sample size of *N* = 200 heavily depended on the proportion of bots (or, again, how many persons actually provided information for the parameter estimates) and model complexity. For small models with *q* = 3 factors, the bias for factor variances and factor loadings was below 7.0% (50% bots); the bias for the correlations increased from 7.0 to 38.8% with increasing proportions of bots. For more complex models with *q* = 6 factors, the a similar pattern could be observed with bias increasing particularly for the factor correlations (9 vs. 21.3% for 10 vs. 25% bots, respectively).

Under the condition of 50% bots, *q* = 6 factors, and *N* = 200, the model broke down with a bias of 438.1, 133.2, and 21.5% for factor variances, factor correlations, and factor loadings respectively. Further inspection (see Figure 1 in the Appendix A) showed that the parameter distribution for factor variances and factor loadings was bimodal with a peak around 0% bias and a second peak around 1,000% bias (variances) or 40% bias (factor loadings). A bivariate distribution (scatter plot) showed an obvious non-overlapping distribution of estimates with and without bias (indicated with red lines). When deleting these “outliers” using a cut-off for the bias of the variance above 200%, the remaining parameters showed unbiased results for factor variances (−2.8%) and factor loadings (4.8%), but still a bias for the factor correlations (114.0%).

For the CFA, Bias was mainly driven by the percentage of bots. Factor variances showed a bias between −15.6 and −12.3%, between −30.4 and −24.6%, and between −49.9 and −39.1% for 10, 25, and 50% bots, respectively. A similar pattern could be observed for factor loadings (and correlations) with a bias between −14.6 and −10.6% (−4.6 and 1.0%), between −28.9 and −24.3% (−13.6 and −4.5%), and between −48.2 and −42.5% (−32.3 and −13.7%), respectively.

### 4.4. Relationship of the Parameter Bias With Random Design Factors

**Figure 2:**
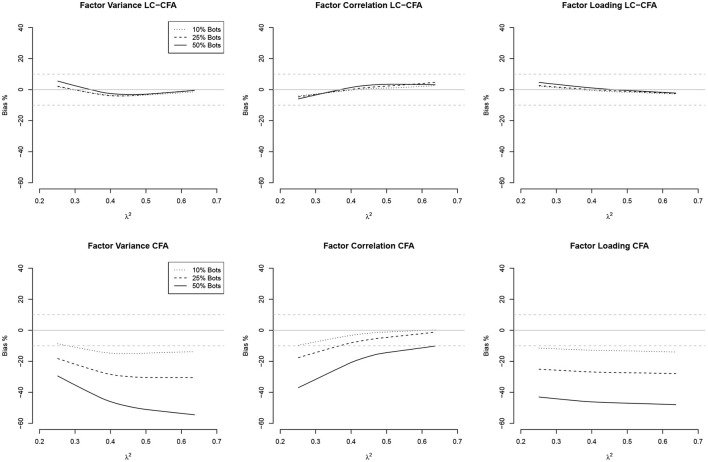
Relationships (loess approximations) between communalities (λ^2^) and the bias of factor variances, factor correlations, and factor loadings from the LC-CFA **(top)** and CFA **(bottom)**. The bias was averaged across conditions of sample size and model complexity.

**Figure 3:**
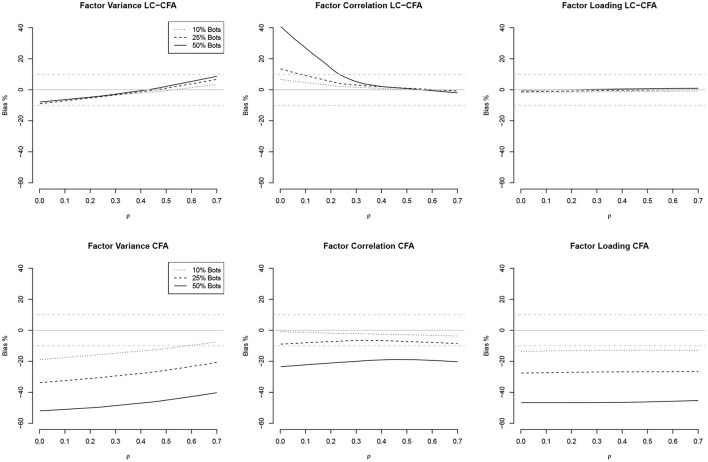
Relationships (loess approximations) between factor correlations (ρ) and the bias of factor variances, factor correlations, and factor loadings from the LC-CFA **(top)** and CFA **(bottom)**. The bias was averaged across conditions of sample size and model complexity.

The relationship between the communalities and factor correlations vs. the bias of factor variances, factor correlations, and factor loadings both from the LC-CFA and CFA are depicted in Figures 2, 3 using loess approximations for each of the conditions of percentage of bots. Results were averaged across the conditions of sample size and model complexity for simplicity and because differences were negligible.

For the LC-CFA, the bias of all three parameter groups did not depend on the communalities. For the CFA, we again observed differences across the percentage of bots as expected. There was an indication that the bias of correlations decreased when the communalities increased; however variances were still underestimated, which increased with higher communalities at least under the condition of 50% bots.

For the factor correlations, we observed a linear relationship for the LC-CFA with the variance bias, which increased with higher amounts of multicollinearity (but was always smaller than ±10%). The factor correlations were overestimated when the actual multicollinearity was low and 50% of the sample consisted of bots. There was a zero-relationship with the factor loading bias. For the CFA, we observed a similar slightly positive relationship with the variance bias, but neither correlations nor factor loadings had a non-zero relationship with the multicollinearity in the data.

### 4.5. Relevance of Predictors in the Latent Class Model

Finally, we investigated how the two predictors for the latent class model performed. Table 4 shows the percentage of significant results (using 95% credible intervals) for the likelihood based person-fit index ϒ_1_ and the nonparametric variability coefficient ϒ_2_. The variability coefficient show 100% significant parameter coefficients across all conditions, that is, it was predictive to distinguish bots and persons. The performance of the person-fit index was suboptimal. For a proportion of 10% bots with a three factor model, the index showed significant estimates between 23.6 and 30.3%. For 10% bots with a six factor model, the index showed only for *N* = 200 a power of 38.4%. For all remaining conditions this power dropped to between 0 and 11.2%. This implied that at least in combination with the variability index the fit index was not sensitive to the detection of bots and had a low power (i.e., few significant prediction in the multinomial logit model).

**Table 4:** Percent significant results for ϒ_1_ (person-fit index) and ϒ_2_ (variability index) in the latent class model of the LC-CFA based on the 95% Credible Interval across conditions of sample size (*N*), number of factors (*q*), and proportion of bots.

**q**	**N**	**ϒ_1_**	**ϒ_2_**
**10% Bots**			
3	200	30.3	100.0
3	400	23.6	100.0
3	800	25.5	100.0
6	200	38.4	100.0
6	400	5.2	100.0
6	800	0.4	100.0
**25% Bots**			
3	200	8.7	100.0
3	400	8.3	100.0
3	800	11.2	100.0
6	200	0.4	100.0
6	400	0.0	100.0
6	800	0.0	100.0
**50% Bots**			
3	200	2.1	100.0
3	400	4.2	100.0
3	800	7.4	100.0
6	200	0.0	100.0
6	400	0.0	100.0
6	800	0.0	100.0

## 5. Empirical Example

To show the efficacy of the LC-CFA for bot identification in an empirical setting we analyzed data obtained from Amazon's Mechanical Turk (MTurk) prior to the implementation of more stringent screening techniques. This data set in particular is useful because bot meta-data was not obscured by IP and geo-location masking approaches that exploiters are now utilizing to remain undetected. Therefore, we established known bots by identifying duplicated geolocations and/or IP addresses. We can thus say with reasonable certainty that the cases flagged are bots, however, the inverse is not true, we will discuss the implications of this in more detail in the discussion.

### 5.1. Data

Data were collected as part of an unrelated experiment in political psychology. Participants (*n* = 395) were recruited on MTurk *via* cloud research (Litman et al., 2017). The dependent measures of this experiment are three commonly used and well validated political ideology measures: an eight item measure of Social Dominance Orientation (SDO; e.g., “Some groups of people are simply inferior to other groups”; Ho et al., 2015), a 10 item measure of Right Wing Authoritarianism (RWA; e.g., “Our country desperately needs a mighty leader who will do what has to be done to destroy the radical new ways and sinfulness that are ruining us”; Rattazzi et al., 2007), and an eight item measure of Nationalism (e.g., “Other countries should try and make their government as much like ours as possible”; Kosterman and Feshbach, 1989). All items were measured on a 7 Likert-type scale (0 = *strongly disagree* to 6 = *strongly agree*).

There was a period of a few months where an increase in automated MTurk responses occurred, which could be identified with identical geolocations. Later it was determined the increase in responses was due to increased activity of a “click farm” which utilized VPN techniques to bypass the studies location requirement (participants were required to be English speaking and live in the United States; Moss and Litman, 2018). These workers completed the surveys in very small time periods in languages foreign to them. The duplicates IP geo-location combination resulted in 159 identified bots, ~40% of the sample. See Appendix B for a descriptive table of the observed data.

### 5.2. Methods

Inverse formulated items (SDO3, SDO4, SD07, and SDO8) were recoded prior to analysis. First, we analyzed the data with the LC-CFA and extracted the predicted classes (bot or not bot). Next, we coded duplicated IP addresses or Geo-locations as known bots. We then computed diagnostic accuracy statistics sensitivity and specificity (Stanislaw and Todorov, 1999), by comparing the estimated latent class (bot or non-bot) to the known bots. Specificity and sensitivity were computed as outlined in the simulation study. In addition, in order to replicate a researcher unaware of bots we then analyze the data with a standard (Bayesian) CFA ignoring bots. We then compared the results of the two models.

For both the LC-CFA and CFA, measurement models were specified in line with the simulation study and existing literature, in that we test a three factor model of SDO, nationalism, and RWA with simple structure. For the LC-CFA, we calculated the variability coefficient as well as the person-fit index and used these as predictors of the latent class (bot or not-bot). Prior distributions and MCMC inputs (e.g., Chain length, burn in, etc.) were specified exactly as described in the simulation study for both LC-CFA and CFA^6^. *Rhat* was used to monitor chain convergence and was calculated identically to that of the simulation study. Models were estimated using JAGS version 4.2 (Plummer, 2003) and deployed in R version 3.6.2 (R Core Team, 2019).

### 5.3. Results

Table 5 contains the standardized factor loadings, factor correlations, and factor variances, as well as the diagnostic measures $\hat{R}$ and ESS for both the LC-CFA and CFA. The LC-CFA exhibited good chain mixing with the highest obtained $\hat{R}<1.01$. We assessed the precision of the posterior estimates with ESS. Zitzmann and Hecht (2019) suggest a practical threshold necessary for summarizing posterior draws of ESS > 400. Both models exhibit ESS estimates for the parameters of interest above this value. In the LC-CFA one parameter (λ_*SDO*5_) was close to the threshold (ESS = 490), however, we are not concerned about the precision of the summary of this posterior distribution. We summarize the posterior with a mean, and as Zitzmann and Hecht (2019) points out, a below optimal ESS has a greater impact on posterior summaries of the distributions tails (e.g., minimum and maximum). It is worth mentioning that the CFA model tended to have ESS values higher than that of the LC-CFA. We believe this is a side effect of the additional parameters in the LC-CFA which leads to slower traversal of the posterior during sampling, in turn resulting in higher auto-correlation in the posterior draws.

**Table 5:** Loading table of LC-CFA and traditional CFA.

**Parameter**	**Factor(s)**	**Item**	**Θ_CFA_**	**Θ_LC-CFA_**	** ${\hat{R}}_{CFA}$ **	**ESS_CFA_**	** ${\hat{R}}_{LC-CFA}$ **	**ESS_LC-CFA_**
**Loadings**								
	**RWA**							
		λ_*RWA*1_	0.91	0.97	1.00	18,000	1.00	740
		λ_*RWA*2_	0.88	0.95	1.00	2,200	1.00	7,900
		λ_*RWA*3_	0.87	0.95	1.00	5,400	1.00	2,200
		λ_*RWA*4_	0.89	0.96	1.00	12,000	1.00	610
		λ_*RWA*5_	0.82	0.93	1.00	18,000	1.00	1,600
		λ_*RWA*6_	0.88	0.96	1.00	7,500	1.00	610
		λ_*RWA*7_	0.90	0.96	1.00	18,000	1.00	4,800
		λ_*RWA*8_	0.91	0.97	1.00	8,900	1.00	2,000
		λ_*RWA*9_	0.88	0.95	1.00	18,000	1.00	6,200
		λ_*RWA*10_	0.91	0.96	1.00	4,800	1.00	2,300
	**SDO**							
		λ_*SDO*1_	0.89	0.94	1.00	4,400	1.00	2,400
		λ_*SDO*2_	0.89	0.93	1.00	6,100	1.00	1,200
		λ_*SDO*3_	0.35	0.90	1.00	3,300	1.00	1,800
		λ_*SDO*4_	0.34	0.90	1.00	10,000	1.00	3,400
		λ_*SDO*5_	0.78	0.88	1.00	18,000	1.01	490
		λ_*SDO*6_	0.86	0.93	1.00	18,000	1.00	1,300
		λ_*SDO*7_	0.30	0.91	1.00	1,900	1.00	4,100
		λ_*SDO*8_	0.40	0.92	1.00	18,000	1.00	8,600
	**NAT**							
		λ_*NAT*1_	0.90	0.97	1.00	2,400	1.00	870
		λ_*NAT*2_	0.82	0.93	1.00	1,500	1.00	980
		λ_*NAT*3_	0.84	0.94	1.00	2,500	1.00	1,800
		λ_*NAT*4_	0.85	0.94	1.00	18,000	1.00	18,000
		λ_*NAT*5_	0.82	0.93	1.00	18,000	1.00	18,000
		λ_*NAT*6_	0.89	0.97	1.00	13,000	1.00	18,000
		λ_*NAT*7_	0.75	0.84	1.00	18,000	1.00	18,000
**Correlations**								
	RWA & SDO		0.77	0.89	1.00	1,500	1.00	1,200
	RWA & NAT		0.76	0.88	1.00	18,000	1.00	1,400
	SDO & NAT		0.90	0.95	1.00	2,500	1.00	18,000
**Variances**								
	RWA		14.44	12.01	1.00	15,000	1.00	4,100
	SDO		12.96	11.10	1.00	4,900	1.00	890
	NAT		11.61	14.40	1.00	4,100	1.00	3,300

*Where Θ are the posterior mean estimates for the associated parameters, $\hat{R}$ provides a descriptive of chain mixing, and the Effective Sample Size is ESS*.

**Figure 4:**
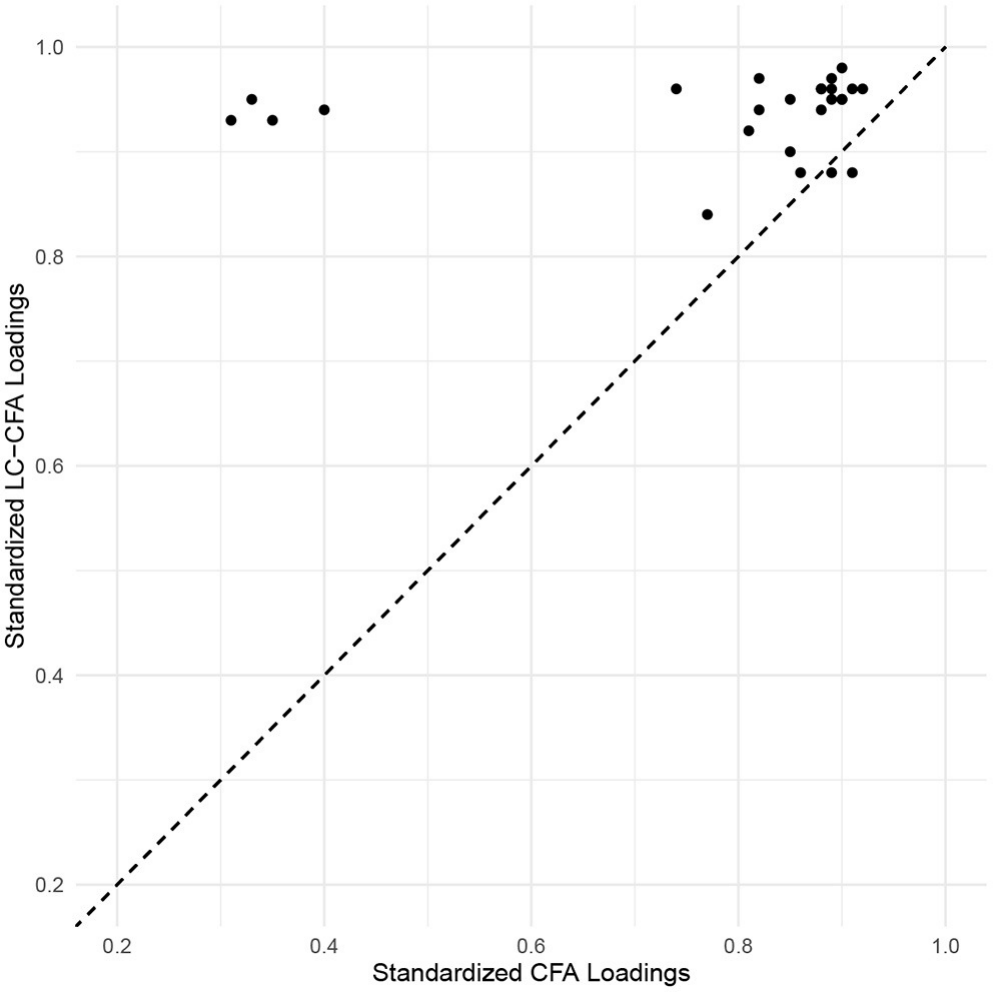
Scatter plot of loading estimates from the LC-CFA and traditional CFA. Values above the diagonal (dashed) line indicates higher standardized loadings for the LC-CFA. The reverse coded items are in the top left part of the plot.

Factor loadings of the LC-CFA were consistently higher than the associated parameters of the traditional CFA. Particularly for the SDO factor, we found comparatively low loadings in the CFA (λ_*SDO*3_ = 0.35, λ_*SDO*4_ = 0.34, λ_*SDO*7_ = 0.30, and λ_*SDO*8_ = 0.40) compared to the LC-CFA (λ_*SDO*3_ = 0.90, λ_*SDO*4_ = 0.90, λ_*SDO*7_ = 0.91, and λ_*SDO*8_ = 0.92); these loadings referred to the only reverse coded items in the survey. Figure 4 provides an illustration of the estimated factor loadings of the LC-CFA (y-axis) vs. the CFA (x-axis).

Factor correlations were also consistently higher in the LC-CFA model (with values ranging from 0.88 to 0.95) compared to the CFA (ranging from 0.76 to 0.90).

With regard to the bot detection in the LC-CFA, we obtained a sensitivity of 71.07% and a specificity was 95.34% (bot classification was designated as the positive identification for computing diagnostic accuracy) with regard to the bots identified with the IP addresses. A contingency table of classification rates is provided in Table 2 (Appendix B). This indicated that we found nearly all bots that were identified with the IP addresses; in addition we classified several persons as bots that showed unique IP addresses. We will discuss this aspect further in the discussion section. With regard to the prediction of the bots with the latent class model, the variability index showed a significant estimate with a credible interval of [−0.423; −0.127]; the person-fit index showed weak predictive power with a credible interval of [−0.017; −0.001].

## 6. Discussion

We believe identification of bots is an important methodological step for online survey data. If this problem is ignored interpretation of any analysis is likely to be biased and in turn replication rates will suffer or interpretations are based statistical artifacts. Our goal was to test and exemplify an approach which could systematically identify bots to improve this issue.

### 6.1. Simulation

The simulation study provided five different important findings. First, we could show that ignoring bots will lead to substantial bias in all models parameter. Factor loadings, factor variances and factor correlations will be severely underestimated, and of course, bias increases with the percentage of bots in the sample. Second, the LC-CFA has a high sensitivity and specificity to identify bots that allowed us to almost perfectly recover all bots under each scenario. Third, using the LC-CFA we could reduce the bias to a degree that can be mostly neglected when sample size was sufficiently large, that is, 400 or more. Smaller sample sizes (*N* = 200) did only provide unbiased estimates if the percentage of bots was not too large and models were not too complex. Fourth, the performance of the LC-CFA did not depend much on the reliability of the items nor the multicollinearity present in the data.

Finally, the variability index outperformed the likelihood based person-fit index in the detection of bots *via* the latent class model. The distinction between the two indices increased particularly with the proportion of bots. One possible explanation is that the increasing amount of bots influences the information contained in the likelihood (see Equation 6). When an increasing amount of persons in the sample does not contribute to the specified model and instead produces more noise, the identification of bots that supposedly show a pattern similar to outliers becomes more complicated (e.g., if 50% of the sample are bots).

### 6.2. Empirical Example

The goal of the empirical example was to exhibit the LC-CFA in a practical setting with known bots. The identification rate of bots in the LC-CFA (specificity = 95.34%) was similar to that found in the simulation study in a comparable condition (*N* = 400 and 50% bots, specificity = 98%). At first it seems the identification of non bots may have suffered (sensitivity = 71.07%) compared to the simulation (*N* = 400 and 50% bots, sensitivity = 99%). However, a potential explanation for this is that some bots may be in the data which were not flagged by the duplicate geolocation approach. Further, it is plausible to assume some responders were inattentive and thus these cases will be classified as bots by the model (e.g., if a person is responding carelessly with random answers). Both of these situations will lead to a reduction in the sensitivity as calculated in this empirical example. Therefore, we feel confident that the obtained sensitivity represent the minimum accuracy of non bot identification and that the *true* accuracy is higher.

A second important finding is that if scales only consist of items that are formulated in the same direction (no inverse formulated items), then ignoring bots may not be as problematic. However, typical recommendations in test construction include the formulation of negatively worded items. In this case, the actual problem (like in the SOD scale here) shows up, for example, with severely biased factor loadings.

### 6.3. Limitations

One of the main limitations in the simulation study was that we did not account for model mis-specification. Two aspects should be addressed here: First, factor models may be mis-specified even for the persons who respond attentively to the questionnaire or the experiment. We did not include this kind of mis-specification and assumed that the general model configuration (which item loads on which factor) were correct. It remains to be investigated how sensitive the bot detection is to such model mis-specifications.

Second, it is likely that as long as inattentive persons use random responses, they will be classified as bots. Even though this changes the interpretation of the class, we think that this is not problematic because inattentive behavior has been shown to contaminate data and bias estimates in a similar fashion as bots (Jin et al., 2018). At this point, there seems to be no reasonable model available that can distinguish bots and careless responders except with very strong and potentially invalid assumptions (regarding response patterns).

### 6.4. Future Directions

While bot identification techniques improve, so do methods to evade detection. First, we expect that programmers might start to provide non-random patterns that mimic actual responses (e.g., other probabilistic functions). Second, click farmers will likely continue to adapt to screening protocols and may begin to employ more deceptive response patterns. In future research, it is necessary to provide methods that are sufficiently general in order to detect bots with different types of fake response patterns.

## 7. Conclusions

We have discussed that bots could be identified with reasonable certainty by flagging duplicate geolocation from survey meta-data. This approach is no longer reliable. In response to the alarms raised in the scientific community, Mturk has implemented filtering methods for known IP geolocation sets. However, this information can be easily obscured by using techniques such as Virtual Private Networks (VPNs) for IP and geolocation spoofing (Pham et al., 2016). Not only are these identity obscuring techniques free, they are readily available, open source, and widely advertised. The LC-CFA approach as we have shown can accurately identify bots in survey data even if survey platforms do not identify them. We are confident that the LC-CFA will be capable of accurate bot identification up until bots can convincingly provide human like response patterns. Therefore, as we have empirically supported its use we recommend using the LC-CFA to improve the quality of data collected from online survey platforms by identifying bots.

## Data Availability Statement

Publicly available datasets were analyzed in this study. This data can be found at: https://www.zacharyroman.com/current-research/latent-class-bot-detection.

## Ethics Statement

Ethical review and approval was not required for the study on human participants in accordance with the local legislation and institutional requirements. The patients/participants provided their written informed consent to participate in this study.

## Author Contributions

ZR is responsible for writing the introduction, discussion, empirical example, conducted the data analysis and tabulation of results as well as final edits, and revisions of the paper. HB conducted the simulation study and also authored the simulation and model specification sections. JM was responsible for the initial political science survey and also authored the description of the data collection and provided theoretical knowledge regarding the magnitude of the bot problem. All authors contributed to the article and approved the submitted version.

## Funding

Open access publishing fees are supported by the University of Tuebingen.

## Conflict of Interest

The authors declare that the research was conducted in the absence of any commercial or financial relationships that could be construed as a potential conflict of interest.

## Publisher's Note

All claims expressed in this article are solely those of the authors and do not necessarily represent those of their affiliated organizations, or those of the publisher, the editors and the reviewers. Any product that may be evaluated in this article, or claim that may be made by its manufacturer, is not guaranteed or endorsed by the publisher.

## Footnotes

^1^In general greater values of the person-fit index suggest greater departures from the factor model, suggesting bot like responses.

^2^Assuming that all items with inverted formulation are recoded accordingly.

^3^The simulation conditions were chosen in part to reflect the factor structure (three factor model) of the empirical example discussed in the next section.

^4^As this prior is relatively informative we conducted a sensitivity analysis with the model described in the empirical example. We re-fit the model under a wide range of Ga prior specifications, from diffuse, to highly informative and misspecified. Posterior means and quantiles varied between runs at a magnitude <0.001% and saw no change in parameter convergence statistics ($\hat{R}$ and ESS). These results suggest little to no influence of the prior on posterior estimates or convergence.

^5^The truncated normal distribution is not necessary. It does not affect the performance of the model in general when compared with un-truncated priors for the factor loadings. In this case, however, it is necessary to check for each factor loading that chain mixing occurred and the sign-switch across chains did not result in a biased interpretation (for similar truncated priors, see e.g., Ghosh and Dunson, 2009).

^6^We felt it was important to replicate common usage, thus we also tested a frequentist CFA with the lavaan package in R. The results were virtually identical, thus, we present only the Bayesian CFA as to provide certainty that the only difference in outcomes is due to the addition of the latent class portion of the model.
