# Supplementary material for: Automated Bot Detection Using Bayesian Latent Class Models in Online Surveys
Source: Front Psychol. 2022 Apr 27;13:789223. doi: 10.3389/fpsyg.2022.789223 (PMC9093679; doi:10.3389/fpsyg.2022.789223)
Supplement: Supplementary file 1 [file Data_Sheet_1.pdf]

Appendix A

Further results for the simulation study

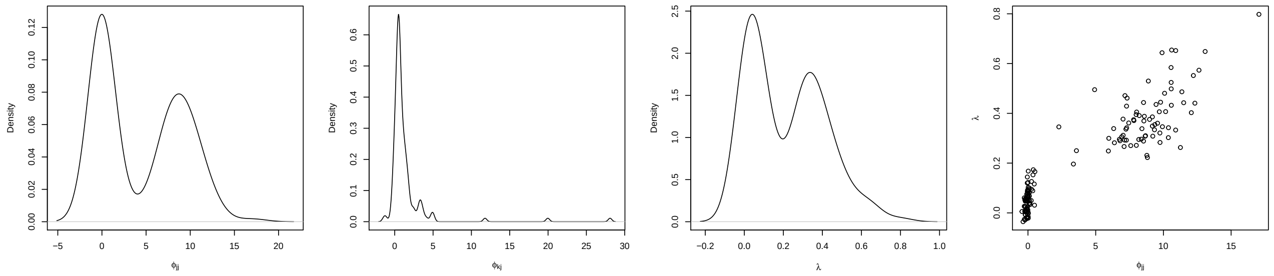

Figure A1. Density plots for the bias of variances (ϕ_{jj}), correlations (ϕ_{kj}), and factor loadings (λ) as well as a scatter plot for the bias of variances vs. factor loadings.

Table A1

Averaged absolute parameter bias of the factor loadings for the LC-CFA and the CFA across conditions of sample size ($N = 200$ and $N = 400$), number of factors (q), and proportion of bots (prop).

N	q	prop	$Bias\|\lambda\|$ LC-CFA	$Bias\|\lambda\|$ CFA
200	3	0.10	10.7	14.8
200	3	0.25	10.5	26.3
200	3	0.50	13.0	43.7
200	6	0.10	9.8	14.1
200	6	0.25	14.2	25.7
200	6	0.50	31.4	43.9
400	3	0.10	7.1	14.4
400	3	0.25	7.5	27.7
400	3	0.50	8.8	46.4
400	6	0.10	7.0	14.2
400	6	0.25	7.6	27.7
400	6	0.50	8.8	46.5
800	3	0.10	5.3	14.7
800	3	0.25	5.6	28.8
800	3	0.50	6.7	47.9
800	6	0.10	5.5	14.8
800	6	0.25	5.8	29.0
800	6	0.50	6.6	48.1

Appendix B

Further results from the empirical example

Table B1

Descriptive statistics of observed Mturk data.

Factor	Item	All Data $\bar{x}$	All Data σ^2	Non-Bots $\bar{x}$	Non-Bots σ^2	Bots $\bar{x}$	Bots σ^2
Right Wing Authoritarianism							
	RWA 1	2.92	1.96	2.92	1.96	5.43	1.38
	RWA 2	2.89	1.87	2.89	1.87	5.47	1.29
	RWA 3	2.90	1.83	2.90	1.83	5.37	1.36
	RWA 4	3.03	2.02	3.03	2.02	5.42	1.28
	RWA 5	3.69	2.02	3.69	2.02	5.60	1.31
	RWA 6	3.38	2.04	3.38	2.04	5.40	1.36
	RWA 7	3.26	1.99	3.26	1.99	5.40	1.40
	RWA 8	3.17	2.10	3.17	2.10	5.44	1.40
	RWA 9	2.89	1.97	2.89	1.97	5.34	1.40
	RWA 10	3.16	2.02	3.16	2.02	5.48	1.31
Social Dominance Orientation							
	SDO 1	2.87	1.91	2.87	1.91	5.43	1.42
	SDO 2	2.58	1.87	2.58	1.87	5.45	1.39
	SDO 3	2.33	1.57	2.33	1.57	2.30	1.01
	SDO 4	2.22	1.44	2.22	1.44	2.26	1.08
	SDO 5	3.36	2.06	3.36	2.06	5.25	1.47
	SDO 6	2.79	2.02	2.79	2.02	5.36	1.37
	SDO 7	2.41	1.56	2.41	1.56	2.33	0.97
	SDO 8	2.10	1.40	2.10	1.40	2.39	1.10
Nationalism							
	NAT 1	3.36	1.99	3.36	1.99	5.48	1.36
	NAT 2	3.65	1.95	3.65	1.95	5.43	1.30
	NAT 3	3.33	1.79	3.33	1.79	5.43	1.26
	NAT 4	3.34	1.84	3.34	1.84	5.45	1.25
	NAT 5	3.56	1.82	3.56	1.82	5.46	1.26
	NAT 6	3.33	1.86	3.33	1.86	5.58	1.23
	NAT 7	3.44	1.85	3.44	1.85	5.60	1.25

$\bar{x}$ is the mean, σ^2 is the standard deviation.

Table B2

Contingency table of Mturk data classification, where bot is the positive classification to be identified.

		Estimated Class	
		Bot	Non-bot
True Class	Bot	113	46
	Non-bot	11	225
